# Volatile Organic Compound Emissions and Human Odor Perception of Dental 3D-Printing Photopolymer Resins

**DOI:** 10.3390/ma19183818

**Published:** 2026-09-08

**Authors:** Wiktoria Kapusta, Mateusz Walerzak, Andrzej Sołtyk, Małgorzata Ponto-Wolska, Artur Winiarski, Leopold Wagner, Łukasz Zadrożny

**Affiliations:** Department of Dental Propaedeutics and Prophylaxis, Faculty of Medicine and Dentistry, Medical University of Warsaw, 02-006 Warsaw, Poland

**Keywords:** photopolymer resins, dental 3D printing, volatile organic compounds, odor perception, indoor air quality, occupational exposure, TVOC

## Abstract

**Highlights:**

• Dental 3D printing photopolymer resins differ significantly in perceived odor intensity.
• Odor perception showed a positive resin-level association with TVOC emissions; however, the relationship was exploratory and not statistically significant.
• A plant-based resin exhibited high odor intensity despite relatively low measured emissions.
• No sex-related differences in odor perception were observed among participants.
• Combining sensory assessment with instrumental monitoring provides complemen-tary information for the evaluation of dental photopolymer resins.

**Abstract:**

The increasing use of photopolymer resins in dental three-dimensional (3D) printing has raised concerns regarding occupational exposure to volatile organic compounds (VOCs). This study aimed to characterize and compare formaldehyde (HCHO) and total volatile organic compound (TVOC) emission profiles of five commercially available dental 3D-printing resins and to explore their relationship with subjective odor perception. Subjective odor perception was assessed during routine educational laboratory activities using a numerical rating scale (0–10). Time-resolved HCHO and TVOC measurements were subsequently performed under standardized chamber conditions using a portable air-quality monitor, with three consecutive 10 min measurement cycles conducted for each resin using the same standardized 200 mL sample. Two participants reporting upper respiratory tract infection symptoms were excluded, resulting in a final sensory sample of 50 participants. Perceived odor intensity differed significantly among the tested resins (Friedman test, χ^2^(4) = 55.54, *p* < 0.001; Kendall’s W = 0.278). A positive resin-level association was observed between mean odor intensity and TVOC concentration at the 10 min endpoint of the first post-agitation measurement cycle (Spearman’s ρ = 0.70); however, this relationship was not statistically significant in the exact two-sided permutation analysis (*p* = 0.233) and was considered exploratory. HCHO–odor correlation analysis was not performed because HCHO concentrations for three of the five resins exceeded the upper measurement range of the instrument. Notably, a plant-based resin exhibited relatively high odor intensity despite comparatively low TVOC emissions, suggesting that total emission magnitude alone may not fully explain odor perception. The tested dental photopolymer resins exhibited distinct material-dependent emission and odor-perception characteristics. Odor perception should therefore be considered complementary to, rather than a substitute for, instrumental emission characterization. Integrating sensory assessment with instrumental emission measurements may support a more comprehensive evaluation of dental photopolymer resins and inform future occupational-safety studies.

## 1. Introduction

Additive manufacturing has become an integral component of contemporary dentistry, with stereolithography (SLA) and digital light processing (DLP) technologies widely employed for the fabrication of dental models, surgical guides, provisional restorations, and orthodontic components [1,2,3,4,5]. These technologies predominantly rely on photopolymer resins, materials valued for their dimensional accuracy, surface quality, and compatibility with digital workflows [6,7,8,9]. However, alongside their increasing clinical and educational use, concerns have emerged regarding occupational exposure to chemical emissions, particularly in laboratory environments where repeated handling of uncured resins is common [10,11,12,13].

Photopolymer resins emit volatile organic compounds (VOCs), including residual monomers, photoinitiator degradation products, and low molecular weight compounds released during storage, agitation, and preparation for printing. Among these substances, formaldehyde is of particular concern due to its classification as a Group 1 carcinogen and its association with mucosal irritation, respiratory symptoms, and neurological complaints [14,15]. Consequently, several studies have focused on instrumental quantification of VOC emissions from 3D printing materials to evaluate their compliance with indoor air quality guidelines and occupational safety thresholds [16]. Because emission behavior depends on resin formulation, comparative characterization of VOC release can provide additional information on material-specific properties of dental photopolymers [13].

Nevertheless, instrumental emission measurements and human sensory perception provide different but potentially complementary information on the behavior of dental photopolymer materials. In educational and laboratory environments, odor perception often constitutes the earliest indicator of airborne chemical presence and may strongly influence perceived safety, comfort, and user behavior, including ventilation practices and compliance with protective measures [17]. Importantly, odor perception is not solely determined by the concentration of emitted compounds but also by their qualitative chemical composition and sensory characteristics [18].

During routine dental laboratory teaching activities, noticeable differences in odor intensity between commonly used photopolymer resins were observed by students. These observations motivated a systematic investigation of whether perceived odor intensity was associated with objectively measured material emission characteristics. Accordingly, anonymized sensory ratings collected during standard educational activities were complemented by controlled instrumental measurements of formaldehyde and total VOC concentrations performed under standardized laboratory conditions.

Therefore, the primary aim of this study was to characterize and compare the HCHO and TVOC emission profiles of five commercially available dental 3D-printing photopolymer resins under standardized laboratory conditions and to explore their relationship with subjective odor perception. By integrating time-resolved instrumental measurements with sensory assessment and non-parametric statistical analysis, this study provides a comparative evaluation of material-dependent emission characteristics and their relationship with perceived odor intensity.

## 2. Materials and Methods

### 2.1. Study Design and Workflow

This study followed a two-stage observational design. The first stage comprised subjective odor-intensity ratings collected during routine educational laboratory activities involving dental photopolymer resins. The second stage comprised controlled, time-resolved instrumental measurements of HCHO and TVOC emissions under standardized laboratory conditions. The combined approach enabled comparative characterization of material-dependent emission profiles and exploratory assessment of their relationship with perceived odor intensity.

### 2.2. Participants

Sensory data were collected from 52 adult second- and third-year dental and dental technology students participating in routine laboratory teaching sessions. All participants were routinely exposed to the evaluated materials as part of their educational curriculum, and no additional exposures or interventions were introduced for research purposes.

Participants were asked to report perceived odor intensity using a numerical rating scale. Two female participants reported symptoms of upper respiratory tract infection at the time of assessment and were excluded due to potential transient olfactory impairment. Final sensory analysis was therefore conducted using data from 50 participants (37 women and 13 men). Individual age data were not collected.

### 2.3. Tested Materials

Five commercially available dental photopolymer resins commonly used in SLA and DLP printing were evaluated:

eSun Water Washable Resin (eSun, Shenzhen, China);

Zirkon Lab Dental Bone Resin (Zirkon Lab, Świdnik, Poland);

Anycubic Plant-Based Resin (Anycubic, Shenzhen, China);

UV Polimer Model One Resin (UV Polimer, Warsaw, Poland);

Anycubic Water Washable Resin (Anycubic, Shenzhen, China).

These materials represent a range of conventional, water-washable, and plant-derived resin formulations.

### 2.4. Subjective Odor Perception Assessment

Odor perception data were collected during routine laboratory activities under supervision. Participants assessed odor intensity by briefly smelling the resins directly from their original containers. Before assessment, the containers were covered with identical opaque paper labels so that the manufacturer and product identity were not visible to participants. The order of the coded resin containers was randomized before assessment to reduce expectancy and systematic order effects. Participants smelled coffee beans between successive resin assessments as a neutral olfactory reference intended to reduce adaptation.

Odor intensity was rated on a numerical scale ranging from 0 (no detectable odor) to 10 (extremely intense odor).

### 2.5. Sample Preparation for Objective Measurements

For each resin, the same standardized 200 mL sample was used throughout the three consecutive measurement cycles. The fixed volume was used to ensure comparable material quantities across all tested resins. Prior to the first measurement cycle, resins were homogenized using a laboratory roller mixer (BFRM-001, Blackfrog, Pruszków, Poland) at 90% rotational speed for 5 min, in accordance with manufacturer recommendations and common laboratory practice. No additional homogenization was performed before the second and third measurement cycles, allowing changes in emission behavior over time after the initial agitation to be observed.

### 2.6. Objective Measurement of VOC Emissions

Objective air-quality monitoring was performed using a Trotec BQ16 air-quality monitor (Trotec GmbH, Heinsberg, Germany). The device measures:

Formaldehyde (HCHO): 0.00–5.00 mg/m^3^ (±5% full-scale accuracy).

Total volatile organic compounds (TVOC): 0.00–9.99 mg/m^3^ (±10% full-scale accuracy). HCHO readings exceeding the upper measurement range of 5.00 mg/m^3^ were displayed by the instrument as over-limit (OL) and were treated as right-censored values rather than exact concentrations.

**Figure 1:**
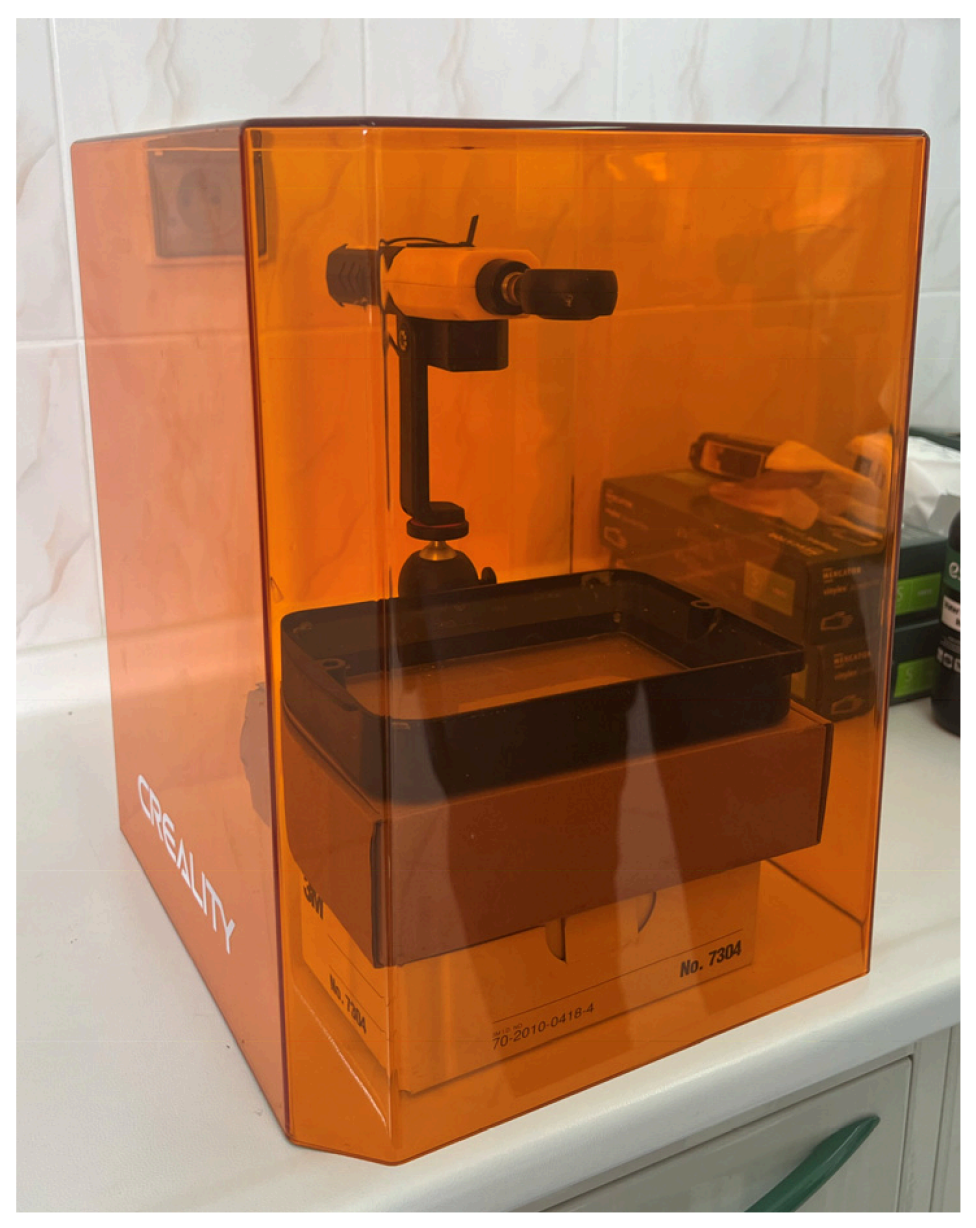
Photographic view of the custom experimental chamber used for objective TVOC and HCHO measurements. The sensor was positioned centrally 15 cm above the resin surface, and the chamber was ventilated between measurement cycles.

Measurements were conducted inside a custom octagonal test chamber with a total height of 36 cm and alternating wall segments of 4.2 cm, 26.6 cm, and 23.2 cm (Figure 1). The sensor was positioned centrally 15 cm above the resin surface. Each resin was monitored during three consecutive 10-min measurement cycles using the same 200 mL sample.

The first cycle was conducted immediately after mechanical agitation, whereas no additional agitation was performed before the second and third cycles. HCHO and TVOC concentrations were recorded at 5 s, 10 s, 15 s, 30 s, and 1 min, and subsequently at 1 min intervals until the 10 min endpoint. The chamber was ventilated between cycles to restore baseline conditions.

### 2.7. Statistical Analysis

Statistical analysis was performed using Microsoft Excel (Microsoft Corporation, Redmond, WA, USA) and SPSS Statistics software (v.29, IBM, Armonk, NY, USA). Descriptive statistics were calculated for subjective odor ratings.

Due to the ordinal nature of the odor intensity scale and repeated-measures study design, differences in odor perception between resins were assessed using the Friedman test. Kendall’s W was calculated as an effect-size measure for the Friedman test. Sex-related differences were analyzed separately for each resin using the Mann–Whitney U test with Holm adjustment for multiple comparisons. The association between mean odor ratings and TVOC concentration was evaluated at the resin level using Spearman’s rank correlation coefficient, with each resin representing one independent observation (*n* = 5). For this exploratory analysis, the TVOC concentration measured at the 10 min endpoint of the first post-agitation measurement cycle was used to provide a standardized comparison of all materials immediately following the same homogenization procedure. Given the very small number of independent observations, statistical significance was assessed using an exact two-sided permutation procedure based on exhaustive enumeration of all 120 possible rank permutations. Resin-level correlation analysis between HCHO concentration and odor perception was not performed because HCHO readings for three of the five materials exceeded the upper measurement range of the instrument, preventing reliable ranking of these concentrations. Statistical significance was set at *p* < 0.05.

### 2.8. Ethical Considerations

Subjective odor perception data were collected during routine educational laboratory activities involving dental photopolymer resins. No additional interventions or experimental exposures were introduced for the purpose of this study. Participants were informed about the sensory assessment and voluntarily agreed verbally to participate and provide anonymous odor-intensity ratings. Blinding of material identity and randomization of presentation order were used solely to reduce assessment bias and did not introduce additional exposure beyond routine educational activities. All data were anonymized prior to analysis and did not contain identifiable personal information. At the time of data collection, the sensory assessment was conducted as part of routine educational laboratory activities, and formal ethics committee review was not sought.

## 3. Results

### 3.1. Study Sample

Subjective odor perception data were initially collected from 52 participants during routine educational laboratory activities. Two female participants (respondents 20 and 21) reported symptoms of upper respiratory tract infection at the time of assessment and were excluded due to the risk of transient olfactory impairment. The final sensory analysis was therefore conducted on 50 participants (37 women and 13 men). Individual age data were not collected.

### 3.2. Differences in Odor Perception Between Resins

Perceived odor intensity differed significantly among the evaluated dental photopolymer resins. Non-parametric repeated-measures analysis revealed a highly statistically significant difference in odor ratings (Friedman test: χ^2^(4) = 55.54, *p* < 0.001; Kendall’s W = 0.278).

The highest mean odor intensity was reported for eSun Water Washable Resin, whereas UV Polimer Model One Resin consistently demonstrated the lowest perceived odor intensity across participants. Zirkon Lab Dental Bone, Anycubic Plant-Based, and Anycubic Water Washable Resins showed intermediate odor ratings. Median values followed the same pattern, with UV Polimer Model One exhibiting the lowest median score.

### 3.3. Sex-Related Differences in Odor Perception

Comparison of odor intensity ratings between female and male participants using the Mann–Whitney U test revealed no statistically significant sex-related differences for any of the tested resins; all comparisons remained non-significant after Holm adjustment for multiple testing (adjusted *p* > 0.05). The present analysis did not identify statistically significant sex-related differences in perceived odor intensity.

### 3.4. Relationship Between Odor Perception and TVOC Emissions

A positive resin-level association was observed between mean odor intensity and TVOC concentration measured at the 10 min endpoint of the first post-agitation measurement cycle. Spearman’s rank correlation coefficient was ρ = 0.70; however, the association was not statistically significant in the exact two-sided permutation analysis (*p* = 0.233) and was therefore considered exploratory.

Resin-level correlation analysis between odor perception and HCHO concentration was not performed because HCHO readings for three of the five resins exceeded the upper measurement range of the instrument, preventing reliable ranking of these concentrations. Notably, the plant-based resin exhibited relatively high perceived odor intensity despite comparatively low measured TVOC concentrations, suggesting that total TVOC concentration alone may not fully explain perceived odor intensity.

### 3.5. Summary of Key Findings

The results demonstrate that:

Dental photopolymer resins differ significantly in perceived odor intensity.

The tested materials exhibited distinct TVOC and HCHO emission profiles across consecutive measurement cycles, with TVOC concentrations decreasing between the first and third cycles for all five resins.

A positive resin-level association between TVOC concentration and perceived odor intensity was observed; however, this relationship was exploratory and not statistically significant.

The HCHO–odor relationship could not be reliably assessed because HCHO readings for three of the five resins exceeded the upper measurement range of the instrument. A summary of the key findings is presented in Table 1.

**Table 1:** Summary of subjective odor perception and 10 min endpoint TVOC and HCHO concentrations for five dental 3D-printing photopolymer resins.

Resin	Mean Odor Intensity (0–10) ± SD	Median Odor Score	TVOC at 10 min, C1/C2/C3 (mg/m^3^)	HCHO at 10 min, C1/C2/C3 (mg/m^3^)	Key Findings
eSun Water Washable Resin	4.44 ± 2.03	4	2.29/2.14/2.03	OL/OL/OL	Highest odor intensity; highest TVOC concentrations
Zirkon Lab Dental Bone Resin	4.10 ± 2.10	4	2.04/1.90/1.81	OL/OL/OL	High odor intensity; HCHO exceeded the measurement range
Anycubic Plant-Based Resin	4.05 ± 1.95	4	0.82/0.72/0.66	0.91/0.72/0.52	Relatively high odor intensity despite comparatively low TVOC concentration
Anycubic Water Washable Resin	3.90 ± 1.84	3.5	2.10/1.76/1.65	OL/OL/OL	Moderate-to- high odor intensity and TVOC concentration; HCHO exceeded the measurement range
UV Polimer Model One Resin	2.20 ± 1.87	1	0.69/0.63/0.60	0.28/0.17/0.12	Lowest odor intensity and lowest measured TVOC and HCHO concentrations

Odor-intensity ratings are presented for the final sensory sample (*N* = 50). C1, C2, and C3 denote three consecutive 10 min measurement cycles performed using the same 200 mL resin sample; mechanical homogenization was performed only before C1. OL—Over Limit; TVOC—total volatile organic compounds; HCHO—formaldehyde; SD—standard deviation. HCHO concentration exceeded the upper measurement range of the instrument (5.00 mg/m^3^) and was therefore treated as a right-censored observation.

## 4. Discussion

The present study investigated the relationship between subjective odor perception and objectively measured volatile organic compound emissions from commonly used dental 3D-printing photopolymer resins. The findings demonstrate significant inter-material differences in perceived odor intensity and a positive but statistically non-significant association between odor perception and TVOC concentration at the resin level, while highlighting the limitations of HCHO measurements exceeding the instrument’s upper measurement range [13,19].

### 4.1. Odor Perception as a Material-Dependent Characteristic

The results of the Friedman test confirmed that odor perception differed significantly among the evaluated resins (χ^2^(4) = 55.54, *p* < 0.001; Kendall’s W = 0.278). UV Polimer Model One Resin consistently exhibited the lowest odor intensity across participants, whereas eSun Water Washable Resin was perceived as the most intense. These findings suggest that odor intensity is a material-specific characteristic that can meaningfully differentiate photopolymer resins beyond their intended mechanical or handling properties [6].

Importantly, no statistically significant sex-related differences in odor perception were identified after Holm adjustment for multiple comparisons. This finding suggests that the observed material-dependent trends were not significantly influenced by participant sex within the studied population.

### 4.2. Relationship Between VOC Emissions and Human Sensory Response

A positive association was observed between perceived odor intensity and TVOC concentration at the resin level (Spearman’s ρ = 0.70). However, this association was not statistically significant in the exact two-sided permutation analysis (*p* = 0.233) and should be interpreted as exploratory because only five independent resin formulations were evaluated. Instrumental air-quality monitoring therefore remains important for objective assessment of emissions in 3D-printing environments [10].

Formaldehyde remains toxicologically important [14]; however, a reliable resin-level relationship between HCHO concentration and odor perception could not be assessed because HCHO readings for three of the five resins exceeded the upper measurement range of the instrument. The exact concentrations and relative ranking of these materials above 5.00 mg/m^3^ were therefore unknown. Odor perception may therefore reflect more than a single measured marker substance [20,21].

### 4.3. Qualitative Composition of VOCs and the Plant-Based Resin Outlier

One of the most notable findings was the behavior of Anycubic Plant-Based Resin, which demonstrated relatively high odor intensity despite comparatively lower measured TVOC concentrations. This discrepancy may reflect differences in qualitative VOC composition or compound-specific odor thresholds; however, this interpretation remains hypothetical because the portable monitor did not identify individual volatile compounds [17].

Resins marketed as “eco-friendly” or plant-derived are often assumed to be less irritating; however, the present data suggest that such classifications do not necessarily translate into reduced sensory impact [22,23,24,25]. Without detailed compound-level analysis (e.g., GC–MS), it cannot be determined which specific volatile components are responsible for this effect, but the observed mismatch underscores the limitations of evaluating resin safety solely on the basis of total emission magnitude [26,27].

### 4.4. Implications for Dental Education and Occupational Safety

In educational and laboratory settings, odor perception may serve as a practical sensory indication of volatile emissions, but it should not be interpreted as a quantitative indicator of toxicological risk or occupational exposure. Strong or unpleasant odors may influence user comfort, alertness, and compliance with ventilation practices [18]. The present findings support the inclusion of sensory perception as a complementary assessment parameter alongside instrumental measurements when evaluating dental 3D-printing materials.

For occupational context, the European Union binding limit values for formaldehyde are 0.37 mg/m^3^ (0.3 ppm) as an 8 h time-weighted average and 0.74 mg/m^3^ (0.6 ppm) as a short-term limit. OSHA specifies a permissible exposure limit of 0.75 ppm as an 8 h TWA and 2 ppm as a 15 min STEL, whereas NIOSH recommends 0.016 ppm as a TWA and a 0.1 ppm 15 min ceiling [28,29,30]. These occupational reference values are not directly comparable with the present chamber measurements, which were obtained 15 cm above an open 200 mL resin sample rather than through representative personal exposure monitoring over the corresponding averaging periods. Therefore, the present measurements should not be interpreted as demonstrating occupational exposure-limit exceedance.

The consistently low odor perception and low measured TVOC and HCHO concentrations observed for the UV Polimer Model One resin suggest that material selection may influence olfactory burden in shared laboratory environments. Conversely, materials associated with strong odor perception may necessitate enhanced ventilation, localized exhaust systems, or procedural adjustments, particularly in teaching laboratories where prolonged exposure may occur [16].

### 4.5. Limitations

Several limitations of the present study should be acknowledged. First, VOC measurements were performed using a portable air-quality monitor rather than compound-specific analytical techniques such as gas chromatography–mass spectrometry, which limits precise identification of individual odor-active compounds [31,32,33]. Consequently, the study could not identify or quantify individual VOCs or determine which specific compounds contributed to the observed odor differences. Second, sensory assessment was based on subjective numerical ratings collected during routine educational activities rather than standardized olfactometric protocols. This approach may introduce variability related to individual olfactory sensitivity, adaptation, and rating behavior and should not be interpreted as a validated olfactometric assessment. Third, the TVOC–odor association was evaluated across only five independent resin formulations, resulting in limited statistical power; the correlation analysis should therefore be considered exploratory. In addition, HCHO concentrations exceeded the upper measurement range of the portable monitor for three of the five materials. These observations did not represent exact concentrations above 5.00 mg/m^3^ and could not be used to determine their relative ordering. Ambient temperature and relative humidity were not systematically recorded. However, all measurements were performed in the same laboratory room under comparable environmental conditions. The lack of continuous temperature and humidity monitoring should nevertheless be considered a limitation, as these factors may influence VOC volatilization.

Additionally, the study population consisted of dental and dental technology students, whose sensory sensitivity and exposure patterns may differ from those of experienced laboratory professionals. Nonetheless, this group represents a relevant population for evaluating material selection and exposure in educational settings.

### 4.6. Future Directions

Future research should combine sensory assessment with more detailed chemical analysis, such as gas chromatography–mass spectrometry (GC–MS), to identify specific VOCs potentially contributing to odor perception and to establish odor thresholds relevant to dental materials. Longitudinal studies assessing cumulative exposure and potential health effects in laboratory environments would further enhance understanding of occupational risks associated with dental 3D-printing resins.

## 5. Conclusions

This study demonstrates that dental 3D-printing photopolymer resins differ significantly in perceived odor intensity and exhibit material-dependent TVOC and HCHO emission characteristics under standardized laboratory conditions. At the resin level, odor intensity showed a positive association with TVOC concentration; however, this relationship was not statistically significant and should be considered exploratory because only five independent resin formulations were evaluated. The relationship between HCHO concentration and odor perception could not be reliably assessed because measurements for three resins exceeded the instrument’s upper HCHO measurement range.

Notably, Anycubic Plant-Based Resin exhibited relatively high odor intensity despite comparatively low measured TVOC concentrations, suggesting that total TVOC concentration alone may not fully explain perceived odor intensity. In contrast, UV Polimer Model One Resin demonstrated the lowest perceived odor intensity and the lowest measured TVOC and HCHO concentrations, indicating that material selection may influence both sensory perception and measured emissions.

No statistically significant sex-related differences in odor perception were identified after Holm adjustment for multiple comparisons. Taken together, these results support the use of sensory assessment as a complementary approach alongside instrumental air-quality monitoring when evaluating dental 3D-printing materials.

From a practical perspective, the findings support attention to appropriate ventilation, handling procedures, and informed resin selection in dental laboratories and teaching facilities.

## Data Availability

The original contributions presented in this study are included in the article. Further inquiries can be directed to the corresponding author.

## Abbreviations

The following abbreviations are used in this manuscript:

3D Printing	Three-Dimensional Printing
VOCs	Volatile Organic Compounds
TVOC	Total Volatile Organic Compounds
SLA	Stereolithography
DLP	Digital Light Processing
